# Clinical differences in monozygotic twins with Rett syndrome: case report and systematic review

**DOI:** 10.1186/s13023-025-03935-6

**Published:** 2025-09-02

**Authors:** Silvia Boeri, Maria Piai, Silvia Russo, Valentina Alari, Francesca Cogliati, Davide Simonetta, Timothy A. Benke, Lino Nobili, Giulia Prato

**Affiliations:** 1 Department of Neurosciences, Rehabilitation, Ophthalmology, Genetics, Maternal and Child Health (DINOGMI), University of Genoa, Genoa, Italy; 2 Unit of Child Neuropsychiatry, IRCCS Istituto Giannina Gaslini, Epicare Network for Rare Disease, Genoa, Italy; 3 Research Laboratory of Medical Cytogenetics and Molecular Genetics, IRCCS Istituto Auxologico Italiano, Milan, Italy; 4 Department of Pediatrics, Neurology and Pharmacology, University of Colorado School of Medicine, Children’s Hospital Colorado, Aurora, CO 80045 USA

**Keywords:** Rett syndrome, Monozygotic twins, MECP2, X chromosome inactivation, Epilepsy

## Abstract

**Background:**

Rett Syndrome (RTT) is a rare, and severe neurodevelopmental disorder that primarily affects females and is primarily (> 96%) due to pathogenic loss-of-function genetic variants of methyl-CpG-binding protein 2 (MECP2). Despite the rarity of the syndrome, sporadic twin cases have been reported. The descriptions have often focused on the phenotype, emphasizing differences or similarities. We report the case of monozygotic (MZ) twins with RTT carrying the same MECP2 mutation and perform a systematic review of the cases of MZ twins.

**Method:**

We searched PubMed and Embase for articles reporting MZ twins with RTT who met Neul criteria and carried mutations in the *MECP2* gene. We focused on phenotypic discordance and X chromosome inactivation (XCI).

**Results:**

Our search yielded 115 results, 18 of which were included in our systematic review. We identified 17 pairs of twins, with 11 showing a discordant phenotype. Data on XCI were reported for only six pairs. We describe MZ twins with typical RTT syndrome who shared the same p.Thr158Met pathogenic variant on the MECP2 gene but exhibited different severity of clinical phenotype, especially regarding epilepsy. The XCI pattern and expression of the wild-type allele in blood were similar in both twins, suggesting that XCI differences assessed in blood may not account for the phenotypic variability. Mononucleate cells were isolated from both twins to generate induced pluripotent stem cells (iPSCs). The patient with more mutated clones presented a more severe phenotype.

**Discussion:**

Cases of MZ twins with RTT are few, and the phenotypic difference described in our case and presented in the literature does not seem to be explained by different XCI patterns. Therefore, more detailed genetic investigations are necessary.

## Background

Rett Syndrome (RTT, MIM 312750) is a rare, severe neurodevelopmental disorder that primarily affects females [1], occurring in approximately 1:10,000 female births [1, 2] and is primarily (> 96%) due to pathogenic loss-of-function genetic variants of methyl-CpG-binding protein 2 (MECP2) [3]. The exact incidence of loss-of-function MECP2 variants in males is unknown but is very rare and found to have a more variable phenotype [4]. MECP2 protein is predominantly expressed in the brain and acts as a transcriptional regulator that activates or inhibits the expression of other genes. Previous studies highlighted that MECP2 contributes to the establishment and maintenance of DNA methylation, therefore MECP2 dysfunction may alter DNA methylation patterns [5]. In addition, RTT severity has been linked to X chromosome inactivation (XCI) [6, 7].

The clinical diagnosis, as described by Hagberg [1], is currently made through the application of specific criteria defined by the Rett Search Consortium in 2010. According to these criteria, RTT is divided into typical and atypical forms [8]. Typical RTT is characterized by a period of regression or loss of hand skills and communication, hand stereotypies and gait abnormalities. Other common features may include intellectual disability, acquired microcephaly, epilepsy (50–90% of cases [9]), movement disorders, dysfunction of the autonomic nervous system, sleep disorders, and scoliosis [8, 10]. RTT is not considered a neurodegenerative disorder and the relationship between disease severity and longevity remains unclear [11]. The syndrome has been described to progress in four stages of the disease: (I) an early stage, with normal birth and psychomotor milestones in the first months; (II) developmental regression with loss of previously acquired motor and/or language skills, starting at the age of 6–18 months; (III) pseudostationary period in which is possible some communicative and cognitive improvement, which may last for years to decades; (IV) late motor deterioration with progressive disability [12]. EEG abnormalities in patients with RTT seemed to follow the progression of stages. A higher percentage of epileptiform activity is observed initially, followed by an increase in amplitude and a general slowing of background activity [13–15]. EEG and evoked potentials correlate with disease severity [16, 17].

RTT is a rare disorder, and even rarer are monozygotic (MZ) twins with this syndrome. The earliest reports of twins with RTT predate the discovery of the MECP2 gene and primarily describe the phenotypic features of the affected people, emphasizing any shared or differing clinical aspects [18–20]. With advancements in genetic knowledge about RTT, researchers have investigated clinical aspects by examining the XCI pattern and the methylation profile of the MECP2 gene to understand differences in twins with RTT [21, 22].

We describe a pair of MZ twins with typical Rett syndrome, both carrying the same pathogenic MECP2 variant. However, the twins exhibited different clinical phenotypes and EEG patterns. To better understand these differences, we analyzed XCI and wild-type allele expression. We then conducted a systematic review to identify cases of MZ twins with clinical diagnosis of RTT or people with pathogenic variants of MECP2, with concordant or discordant phenotypes; people with pathogenic variants in *CDKL5* [23] and *FOXG1* [24] (historically considered to be in association with RTT prior to genetic and phenotypic correlations) were not included.

## Methods

### Patient selection

We selected the MZ twins with RTT carrying the same pathogenetic variant of the MECP2 gene (p.Thr158Met) from the internal database of the Child Neuropsychiatry Unit of Giannina Gaslini Institute. Further genetic investigations were performed according to the 1964 Declaration of Helsinki and after obtaining informed parental consent provided by the Gaslini Institute.

### Genetic analysis

XCI was investigated by segregation analysis of Humara Androgen Receptor polymorphism [25, 26]. Each twin’s cDNA was sequenced to understand whether each case’s wild-type or mutated allele was equally or oppositely expressed [27]. Mononucleate cells were isolated from both twins to generate induced pluripotent stem cells (iPSCs) (according to the protocol described in Perego et al., 2022) [27]. In order to confirm the genomic stability of the IPSCs clones, karyotype and molecular karyotype were performed (Infinium CytoSNP-850 K-Illumina).

### Literature search strategy

The systematic search was performed by two authors independently (SB and SR), according to PRISMA 2020 guidelines. The search was conducted on two databases (PUBMED and EMBASE) in April 2024. Articles were searched for MZ twin cases diagnosed with RTT from 1986 to 2023. The search of the databases used the following keywords: (Rett Syndrome OR Rett OR RTT) AND (twin). The following eligibility criteria were used:

- Inclusion criteria: patients with a clinical diagnosis of Rett syndrome according to Hagberg’s descriptions [1] and/or Neul’s 2010 criteria [8], MECP2 molecular diagnosis, female MZ twins described in case reports, reviews, case series, and English language texts.
- Exclusion criteria: “Rett-like diagnosis” (i.e., lack of details to ascertain meeting RTT criteria), patients carrying other genetic mutations and descriptions of heterozygous twins.

The two authors blinded extracted clinical information, and any disagreements were resolved by an additional reviewer (GP).

## Case report

We report on 27-year-old MZ female twins with typical RTT syndrome at clinical stage IV associated with *de novo* p.Thr158Met variant of the MECP2 gene, known to be associated with a severe phenotype [28].

They were second born of healthy non-consanguineous parents. The uneventful pregnancy was monochorionic and diamniotic, and they were born by normal delivery at the 32nd week of gestation. The twins had birth weights of 1470 g (twin A) and 1540 g (twin B) respectively, and both presented with generalized hypotonia at birth. Head circumference at birth was not available. Microcephaly has been reported in both twins, but there are no measurements available for early life.

They both showed delayed acquisition of milestones with independent walking at 18 months (characterized by wide gait) and no acquisition of sphincter control. Speech development was impaired: twin A never acquired verbal language, while twin B initially acquired one single word, but then showed regression with complete loss of use. By the age of twelve months, they both showed regression of relational skills: twin A had a greater impairment, with poorly modulated eye contact and less communicative intentionality, while twin B retained discreetly modulated eye contact and greater use of vocalizations with communicative intent. They both showed the appearance of two-manual hand-washing motor stereotypies with consequent gradual impairment of functional use of the hands. Twin A lost the ability to manipulate objects, while twin B retained a minimal degree of manipulation (such as touching food or holding objects for a few seconds). On the Hand-Apraxia Scale, twin A obtained a score of zero, while twin B had a score of 2 (best function).

Both twins maintained the ability to walk with support, though twin A had greater lower limb spasticity. At the age of 2, they both underwent a brain magnetic resonance imaging (MRI) that was interpreted as normal.

Twin A also developed bruxism, oropharyngeal dysphagia, mild scoliosis (Cobb angle = 15), constipation requiring laxatives with benefit, important air-swallowing resulting in abdominal bloating, and worsening of spasticity, with partial tendon retraction of the lower limbs and limitation of independent walking (she took a few steps with assistance). Twin B exhibited milder bruxism and spasticity, rare episodes of dysphagia, but worse scoliosis (Cobb angle = 30), treated conservatively with a rigid corset, and constipation requiring laxative therapy with limited effect. Moreover, twin A showed sleep-wake cycle disorder characterized by frequent nocturnal awakenings with episodes of apnea-hypopnea during sleep, sometimes associated with desaturation of less than 10 s (SpO2 from 95 to 85% documented with polysomnography) that did not require intervention. Twin A showed episodes of awake hyperventilation and respiratory pauses without oxygen desaturation. Twin B only had mild awake breathing disturbances with evidence at polysomnography of respiratory pauses without oxygen desaturation both in wakefulness and sleep. (Table 1)

**Table 1:** Clinical and behavioral differences between twins

	Twin A	Twin B
Non-verbal Communication	3	2
Verbal Communication	3	2
Scoliosis	1	2
Deambulation	3	2
Sleep disorders	3	1
Epilepsy	2	1
Breathing problems	3	1
Muscular tension	3	1
Gastrointestinal problems	2	3

1: mild, 2: moderate, 3: severe

**Fig. 1:**
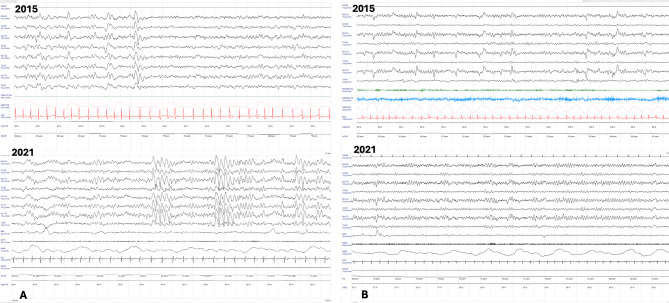
Polygraphic recording: EEG SI 10–20 (8 electrodes), respiration, electrocardiogram, electromyogram (mylohyoid, extensor digitorum, opponens pollicis muscles), electrooculogram (only in the 2021 recording), oxygen saturation, heart rate. Parameters EEG: 30 s/pg, 10 microV/mm, high-pass filter 0.3 s, low pass-filter 15 Hz, Notch 50 hz. **A.**Top (2015): Wake EEG. Slow generalized low voltage background activity in the theta rhythm (4 Hz). Generalized epileptiform slow sharp-waves, mainly expressed in the fronto- central derivations. Respiration: bradypnea and respiratory pauses of 3–5 s without oxygen desaturation (SpO2 98–99%). Bottom (2021): Wake EEG. Slow generalized background activity, high voltage spike-waves and waves (2–3 Hz) organized in brief runs more represented in the fronto-central regions bilaterally. Respiration: irregular with hypopneas without oxygen desaturation (SpO2 98–99%). **B.**Top (2015): Wake EEG. Slow background activity in the theta rhythm. Isolated sharp waves on the fronto-central derivations bilaterally. Regular respiratory pattern, oxygen saturation is stable (99–100%). Bottom (2021): Wake EEG. Background activity in the theta-delta rhythm with interposed isolated low-voltage slow waves on the fronto-temporal derivations bilaterally. Irregular respiration with respiratory pauses, but oxygen saturation is stable

Both twins developed epilepsy at the age of 4 years, characterized by the onset of tonic-clonic seizures. The twins exhibited epileptic and EEG features over the years consistent with Lennox- like syndrome. In twin A, epilepsy had a more severe course, with the development at the age of 11 years of resistance to anti-seizure medications (ASMs) and persistence of multiple daily tonic-clonic seizures, only partially controlled by the combination of two ASMs (carbamazepine –CBZ and phenobarbital –PHB). After the pubertal stage, seizures were mainly tonic and with reduced frequency (weekly). The electroencephalogram (EEG) showed a progressive breakdown of background activity, which was replaced by monomorphic theta rhythms of medium voltage; in addition, slow spike-waves (2–3 Hz) of high voltage were present in bilateral fronto-central regions. (Fig. 1A) In twin B, epilepsy reached good seizure control with CBZ monotherapy. As in her sister, the seizures were reduced in the post-pubertal phase, occurring at a monthly/bimonthly frequency. Background and epileptiform EEG activity remained quite stable over the years. (Fig. 1B) The Rett Assessment Rating Scale (RARS) [29] at 27 years showed high severity for twin A (score 88) and moderate severity for twin B (score 80.5).

**Fig. 2:**
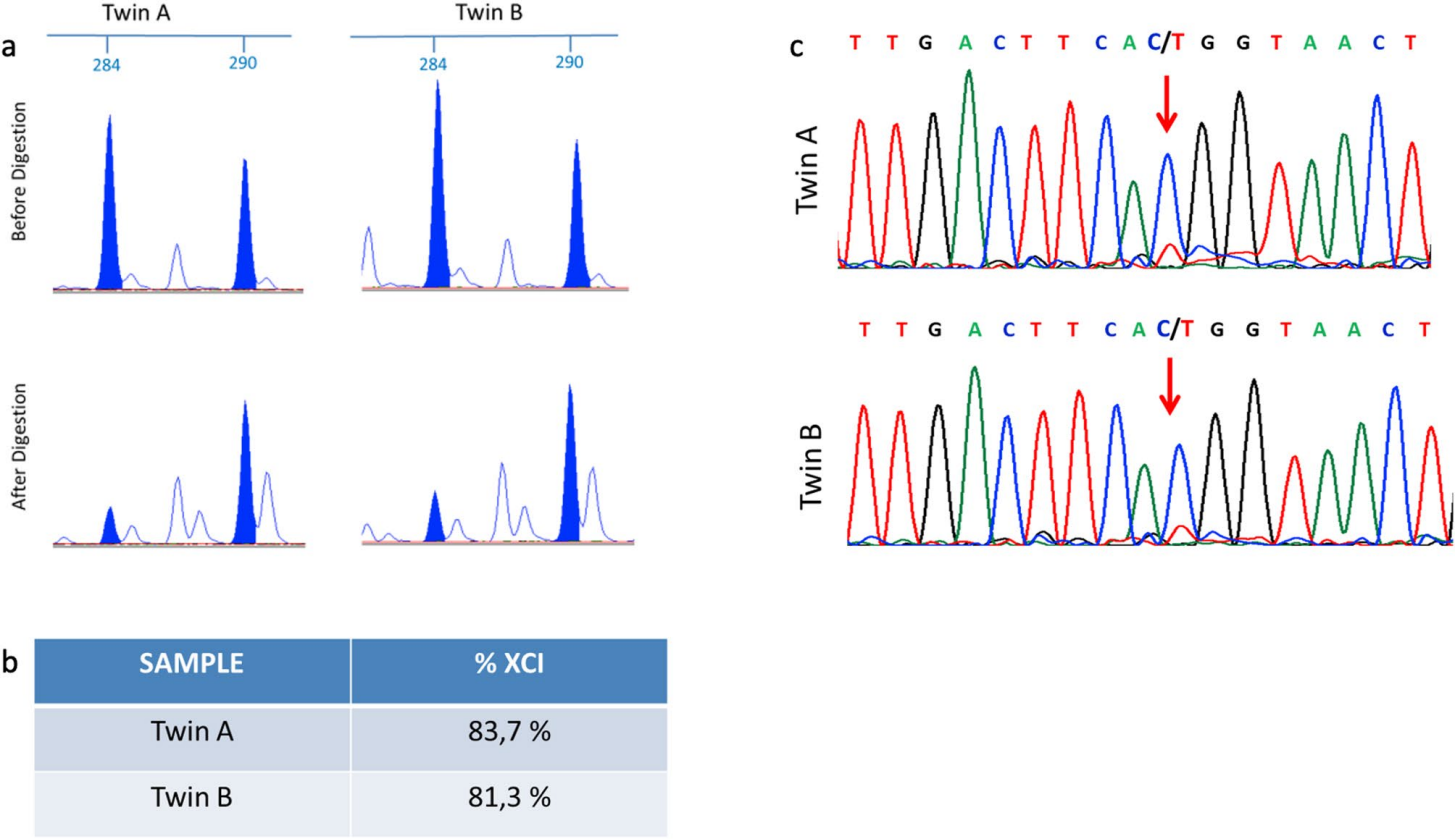
XCI inactivation and unbalanced expression of the MECP2 alleles: (**a**) electropherogram profile of AR locus in both the twins is identical, either in genomic DNA and HhaI digested DNA, (**b**) shows the percentage XCI (normal values 20–80%), (**c**) electropheragram of cDNA sequencing showing the unbalanced expression of MECP2 variant NM_ 004992.4:c.473 C > T, p.(Thr158Met) identical in the two girls

At the age of 6, the girls underwent DNA sequencing by single-gene testing, revealing in both the MECP2 *de novo* variant NM_004992.4:c.473 C > T, p.(Thr158Met) a hot-spot mutation associated with typical RTT. Discrepancies in their clinical phenotypes led to an investigation of XCI patterns and wild-type allele expression, revealing borderline XCI patterns (83% for twin A and 81% for twin B) and predominantly wild-type allele expression in blood (Fig. 2A and B). Mononucleate cells were isolated from both twins to generate iPSCs according to the protocol described by Perego et al. [27]. Four clones were obtained from twin A and 12 clones from twin B. Each clone was investigated for the expression of the wild-type or mutated allele. Due to XCI, each clone expresses only the mutated or the wild-type allele. cDNA sequence of the region including our variant revealed the occurrence of 1/4 clones expressing the MECP2 pathogenetic variant in twin A, and 0 out of 12 clones in twin B. (Table 2)

**Table 2:** Expression of wild-type and mutated clones in the iPSCs from twin A and twin B

Patient	Wild Type clones	Mutated clones	Both the alleles expressed
Twin A	2	1	1
Twin B	11	0	1

These findings suggest that the proportion of cells expressing the mutated MECP2 allele differs between the twins, with twin A likely having a higher number of affected cells. Given the crucial role of MECP2 in the central nervous system, this imbalance may contribute to differences in disease severity between the two siblings.

### Systematic review

**Fig. 3:**
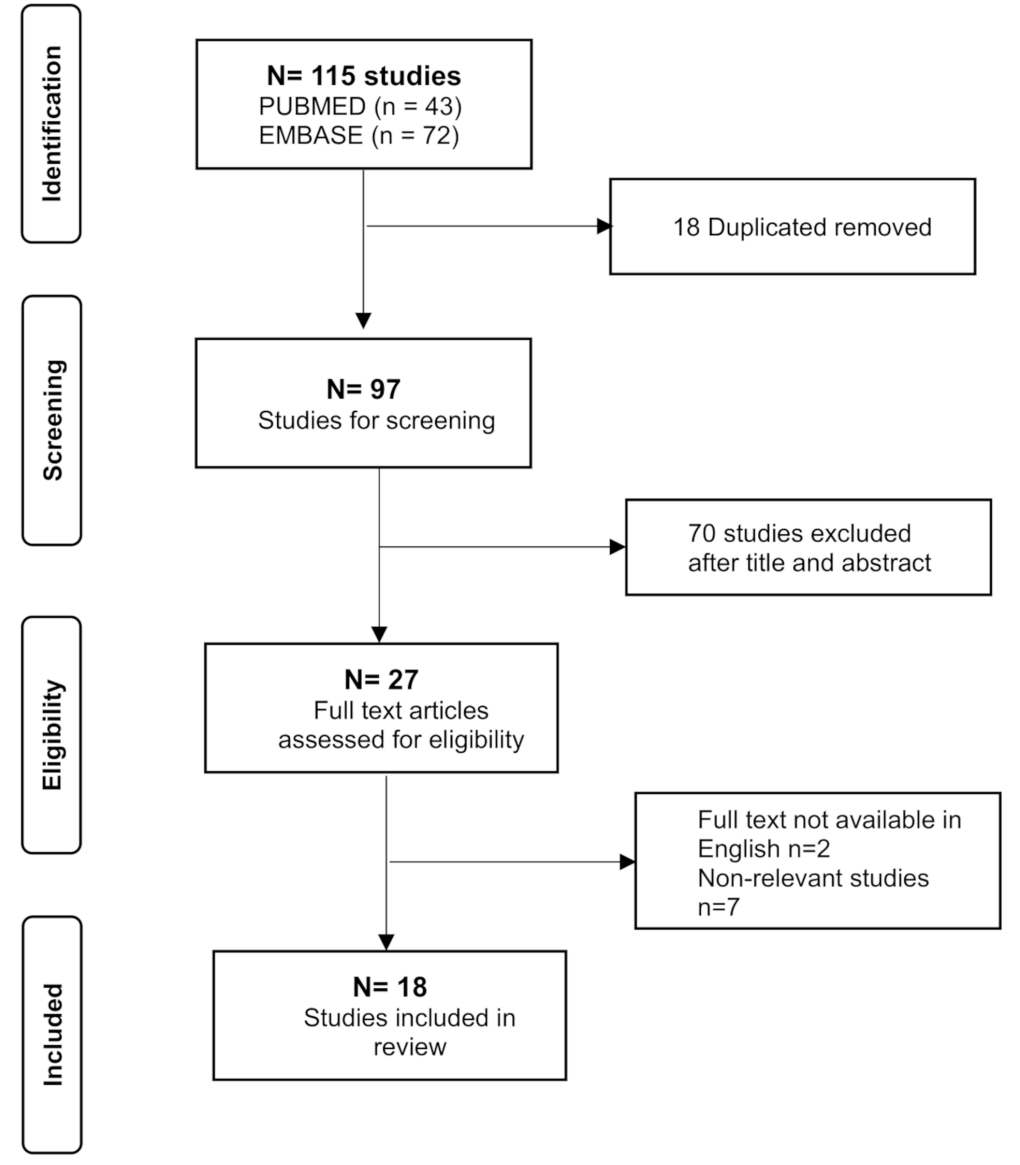
PRISMA flowchart for included studies

Using PRISMA guidelines, we identified 115 articles; removing duplicates we screened 97 papers. We subsequently excluded 70 papers based on title and/or abstracts that were not relevant to our review. We then excluded 9 papers as non-English language or non-relevant. (Fig. 3) We included 18 articles in our final analysis. (Table 3) In Table 3, we included MZ twin pairs with RTT described in the literature focusing mainly on concordant or discordant phenotype and the degree of XCI.

**Table 3:** Phenotypic concordance or discordance, MECP2 variant, X chromosome inactivation in MZ RTT twins previously reported and in our case

CITATION	*N*° OF COUPLES	PHENOTYPE		XCI	PARENTAL ORIGINE ACTIVE CHR	MECP2 VARIANT
Hagberg B, Witt-Engerstöm, 1986	**3**	NA	NA	NA	NA	NA
Coleman M, 1987, Coleman M, 1988	**1**	**D**	(slightly different in hand stereotypies, epilepsy)	BL (30:70)	Mother	NA
Tariverdian1987; Tariveridian 1990	**1**	**C**	concordant	NA		NA
Partington MW 1988	**1**	**D**	Different in ambulation, epilepsy, and breathing	NA		NA
Bruck I 1991	**1**	**D**	Different in epilepsy			
Migeon 1995	**2** (+ 1 described in Coleman)	**C**		Random	Both	NA
		**D**	for phenotype (see Coleman)	Tot Skewed BL (30:70)	Mother	NA
		**D**	One twin is healthy RTT twin	Random	Both	NA
Subramaniam, 1997 (twin described in Migeon)	See: Migeon					
Ogawa A, 1997; Ishii T, 2001	**1**	**D**	Different in adolescence for seizures, scoliosis hand stereotypies	88% worst 52%better	Mother	p.Arg294Ter
Cheadle J, 2000	**1**	**D**	Different in severity of developmental disturbance	NA	NA	p.Arg255*
Carter 2008	**1**	**D**	Different in GM volumes (frontal, parietal, temporal) and language delay	Random		c.1160 (del26)
Mittal K 2011	**1**	**D**	Slightly discordant in spasticity, language and Vineland Social Maturity Scale (19 vs. 23)	NA		Deletion exon3 only
Zhang X, 2012	**1**	**D**	1 RTT atypical and 1 congenital	not specified		not specified
Kunio M, 2013	**1**	**D**	age of regression (6 month vs. 2,5y), Clinical score (7 vs. 27)	Random (studied blood, hair, fibroblast)	Both	p.Gly269 AlafsX288
Einspieler C, 2014	**1**	**D**	Hand stereotypies, scoliosis, ambulation and communication	NA		NA
Titlestad & Eldevik, 2019	**1**	**D**	Slightly discordant (difference in scoliosis)	NA		NA
***This Study***	**1**	**D**	***Different in epilepsy***,*** hand use***,*** spasticity***	***BL 83%*** ***and 81%***	***Healthy allele***	***p.Thr158Met***

Abbreviation: D: discordant, C: concordant, XCI: X chromosome inactivation, GM: grey matter, NA: not available

Seventeen pairs of MZ twins with RTT have been described in the literature. (Table 3)

In our review, we reported all works containing cases of MZ twins; however, some descriptions of twins were further described in other papers and were not added to the total number of pairs. The first three cases of MZ twins date to 1986; however, no phenotypic characteristics of the patients are further reported in Hagberg’s article [30]. Eleven papers report only the phenotypic features of the patients without referring to genetic investigations (XCI, methylation of MECP2 gene): ten papers precede the discovery of the MECP2 gene (year 1999) so the diagnosis of RTT is exclusively based on clinical criteria [18–20, 30–36].

Three case reports following the discovery of the relationship of MECP2 to RTT do not report the pathogenetic variant [37–39] but are based exclusively on similarities or clinical differences between twins. In six pairs, XCI is reported: in four pairs the inactivation was random [21, 33, 40], while in two pairs it was skewed with more active maternal or paternal allele [18, 31, 33, 36, 41]. (Table 3) Among the twins with random XCI, three pairs showed a discordant phenotype [21, 33, 40], while in one the phenotype was concordant [33]. Both pairs with skewed XCI presented with discordant phenotypes [18, 31, 33, 36, 41]. (Table 3)

Eleven twin pairs (multiple authors have described the same pair of twins) [18, 20–22, 31, 33, 34, 36–42] show discordant or mildly discordant clinical features mainly concerning development, hand stereotypies, and ambulation; only in three pairs differences between epilepsy features are described [18, 20, 31, 33, 34]. Phenotypic differences and the pattern of XCI are fully described in Table 3.

## Discussion

Described cases of MZ twins with RTT in the literature (Table 3) are rare and mainly focused on clinical aspects. We provide a description of twins that includes a complete description of the clinical differences in epilepsy features. In the literature, most MZ RTT twins are slightly or significantly discordant in phenotype and clinical evolution; phenotypic differences are related to age of regression, movement, behavior, and hand stereotypies. In a single pair of twins, the difference is about the presence or absence of clinically diagnosed RTT: Migeon et al. described a twin pair that included a girl with a nearly normal phenotype and her twin with RTT (however, the MECP2 mutation status was not reported) [33]. Our review reveals 11 pairs of MZ twins with different characteristics not only clinically but also neuroanatomically: differences in gray matter volume between the twins are highlighted in the studies of Subramaniam et al. and Carter et al. [35, 40].

Epilepsy and EEG tracing were rarely investigated and studied. Coleman and Kunio described a twin pair with discordant epileptological pictures but limited references to EEG pattern [18, 21]. Bruck et al. described a twin pair, in which one sister showed well-controlled seizures with ASM monotherapy and an EEG characterized by epileptiform abnormalities in the temporal region, while the other sister had only partial seizure control on ASM bi-therapy and an EEG with epileptiform abnormalities in the centrotemporal region [34]. In Partington’s case report, the presence of generalized type seizures in twins is only briefly mentioned [20].

In our case, the main phenotypic differences are related to epilepsy. Twin A developed drug-resistant epilepsy, poorly controlled by two ASMs (CBZ and PHB), while her sister showed a good response to ASM monotherapy (CBZ). The EEG pattern was also discrepant. In twin A EEG tracing had more epileptiform abnormalities in addition to the classic monomorphic slow waves shared with her sister. (Fig. 1) Twin A with the more severe epilepsy showed a worsening over time of background activity and epileptiform abnormalities in the EEG tracing, while the other showed a pattern that was more stable over time. We reported the evolution of the EEG trend. (Fig. 1) Evaluation of other aspects typically involved in RTT also revealed a more severe global clinical picture in twin A, with worse sleep-wake rhythm, respiratory disturbances (prevalent in sleep), and a greater degree of spasticity with impaired gait. (Table 1)

To better understand the underlying mechanism from a genetic point of view, however, the X inactivation chromosomal pattern was interrogated to explain the discrepancy in phenotype with a different expression of the pathological allele. These phenotypic differences have also been described in previous works, and in three of these, it is possible to find a possible explanation for these differences in the different patterns of XCI [18, 33, 41], which was not detected in our patients.

Ishii and colleagues investigated the possible genotypic differences in phenotypically dissimilar MZ RTT twins: in their work, the phenotypic differences subtended a different pattern of XCI. In particular, the twin with the most severe picture showed a higher expression of active paternal allele compared with the other [41]. In Migeon’s twins, a complete skewing motivates the finding of a total discrepancy, but in Kunio et al. the study of various tissues showed a random XCI in all of them [21, 33]. The Ishii paper hypothesized a preferred XCI of the MECP2 of the paternal allele, according to previous studies and the more recent paper of Fang et al. [7, 43, 44]. In our study, identically the girls had borderline XCI values, and their cDNA expressed preferentially the wild-type allele. Investigation of iPSCs from our twins confirmed the prevalent expression of the normal allele. However, the presence of a higher percentage of mutated clones in the twin with a more severe phenotype gives rise to the occurrence of a major number of cells expressing MECP2 in twin A also in the brain. Should we confirm, replicating the experiments, that in blood there are more cells expressing the pathogenic variant of MECP2, we could hypothesize that the twins have consistent tissue mosaicism and that the mutated allele may be expressed only in target tissues.

The increased expression of the normal allele in the blood had already been observed in RTT patients with MECP2 mutations and seems to be associated with more severe phenotypes [27, 45]. We hypothesize that this may represent a sort of protective mechanism, as the expression of mutated alleles throughout the body would be too devastating. Further investigations on other tissues, such as fibroblasts, and a fair number of patients are needed to confirm this hypothesis. The fact is relevant if we consider the future option of X chromosome reactivation as a possibility of therapy.

## Conclusion

In conclusion, we describe a pair of RTT twins, with the same genotype and different phenotypes, both clinically and in EEG pattern. The clinical differences described in our paper could thus be explained by other genetic mechanisms, such as different expressions of the mutated allele in the nervous tissues, the presence of epimutations, or the presence of pre-twinning mutations, which are difficult to recognize. We believe that this work, along with the review of the cases described so far, allows us to deepen our knowledge of the clinical aspects of twins with RTT syndrome, particularly with regard to epilepsy, and to focus attention on the variability of allelic expression of the MECP2 gene in patients with a very severe phenotype, which needs, however, further, and more in-depth genetic investigations.

## Data Availability

The datasets used and/or analysed during the current study are available from the corresponding author on reasonable request.

## Abbreviations

RTT: Rett syndrome
MECP2: methyl-CpG-binding protein 2
MZ: Monozygotic
XCI: X Chromosome inactivation
iPSCs: Induced pluripotent stem cells
MRI: Magnetic resonance imaging
ASM: Antiseizure medication
CBZ: Carbamazepine
PHB: Phenobarbital
EEG: Electroencephalogram

## References

[1] Hagberg B, Aicardi J, Dias K, Ramos O. A progressive syndrome of autism, dementia, ataxia, and loss of purposeful hand use in girls: rett’s syndrome: report of 35 cases. Ann Neurol. 1983;14:471–9. 10.1002/ana.410140412. 6638958 10.1002/ana.410140412

[2] Petriti U, Dudman DC, Scosyrev E, Lopez-Leon S. Global prevalence of Rett syndrome: systematic review and meta-analysis. Syst Rev. 2023;12:5. 10.1186/s13643-023-02169-6. 36642718 10.1186/s13643-023-02169-6 PMC9841621

[3] Amir RE, Van den Veyver IB, Wan M, Tran CQ, Francke U, Zoghbi HY. Rett syndrome is caused by mutations in X-linked MECP2, encoding methyl-CpG-binding protein 2. Nat Genet. 1999;23:185–8. 10.1038/13810. 10508514 10.1038/13810

[4] Neul JL, Benke TA, Marsh ED, Skinner SA, Merritt J, Lieberman DN, et al. The array of clinical phenotypes of males with mutations in Methyl-CpG binding protein 2. Am J Med Genet B Neuropsychiatr Genet. 2019;180:55–67. 10.1002/ajmg.b.32707. 30536762 10.1002/ajmg.b.32707 PMC6488031

[5] Marano D, Fioriniello S, D’Esposito M, Della Ragione F. Transcriptomic and epigenomic landscape in Rett syndrome. Biomolecules. 2021;11:967. 10.3390/biom11070967. 34209228 10.3390/biom11070967 PMC8301932

[6] Merritt JK, Fang X, Caylor RC, Skinner SA, Friez MJ, Percy AK, et al. Normalized clinical severity scores reveal a correlation between X chromosome inactivation and disease severity in Rett syndrome. Genes (Basel). 2024;15. 10.3390/genes15050594. 10.3390/genes15050594 PMC11120815 38790223

[7] Fang X, Butler KM, Abidi F, Gass J, Beisang A, Feyma T, et al. Analysis of X-inactivation status in a Rett syndrome natural history study cohort. Mol Genet Genomic Med. 2022;10:e1917. 10.1002/mgg3.1917. 35318820 10.1002/mgg3.1917 PMC9034674

[8] Neul JL, Kaufmann WE, Glaze DG, Christodoulou J, Clarke AJ, Bahi-Buisson N, et al. Rett syndrome: revised diagnostic criteria and nomenclature. Ann Neurol. 2010;68:944–50. 10.1002/ana.22124. 21154482 10.1002/ana.22124 PMC3058521

[9] Pintaudi M, Calevo MG, Vignoli A, Parodi E, Aiello F, Baglietto MG, et al. Epilepsy in Rett syndrome: clinical and genetic features. Epilepsy Behav. 2010;19:296–300. 10.1016/j.yebeh.2010.06.051. 20728410 10.1016/j.yebeh.2010.06.051

[10] Fu C, Armstrong D, Marsh E, Lieberman D, Motil K, Witt R, et al. Multisystem comorbidities in classic Rett syndrome: a scoping review. BMJ Paediatr Open. 2020;4:e000731. 10.1136/bmjpo-2020-000731. 33024833 10.1136/bmjpo-2020-000731 PMC7509967

[11] Neul JL, Benke TA, Marsh ED, Suter B, Fu C, Ryther RC, et al. Clinical features and disease progression in older individuals with Rett syndrome. Genes (Basel). 2024;15. 10.3390/genes15081107. 10.3390/genes15081107 PMC11353339 39202466

[12] Hagberg B. Clinical manifestations and stages of Rett syndrome. Ment Retard Dev Disabil Res Rev. 2002;8:61–5. 10.1002/mrdd.10020. 12112728 10.1002/mrdd.10020

[13] Portnova G, Neklyudova A, Voinova V, Sysoeva O. Clinical EEG of Rett syndrome: group analysis supplemented with longitudinal case report. J Pers Med. 2022;12:1973. 10.3390/jpm12121973. 36556193 10.3390/jpm12121973 PMC9782488

[14] Tarquinio DC, Hou W, Berg A, Kaufmann WE, Lane JB, Skinner SA, et al. Longitudinal course of epilepsy in Rett syndrome and related disorders. Brain. 2017;140:306–18. 10.1093/brain/aww302. 28007990 10.1093/brain/aww302 PMC5278305

[15] Glaze DG, Percy AK, Skinner S, Motil KJ, Neul JL, Barrish JO, et al. Epilepsy and the natural history of Rett syndrome. Neurology. 2010;74:909–12. 10.1212/WNL.0b013e3181d6b852. 20231667 10.1212/WNL.0b013e3181d6b852 PMC2836870

[16] Saby JN, Benke TA, Peters SU, Standridge SM, Matsuzaki J, Cutri-French C, et al. Multisite study of evoked potentials in Rett syndrome. Ann Neurol. 2021;89:790–802. 10.1002/ana.26029. 33480039 10.1002/ana.26029 PMC8882338

[17] Saby JN, Peters SU, Benke TA, Standridge SM, Swanson LC, Lieberman DN, et al. Comparison of evoked potentials across four related developmental encephalopathies. J Neurodev Disord. 2023;15:10. 10.1186/s11689-023-09479-9. 36870948 10.1186/s11689-023-09479-9 PMC9985257

[18] Coleman M, Naidu S, Murphy M, Pines M, Bias W. A set of monozygotic twins with Rett syndrome. Brain Dev. 1987;9:475–8. 10.1016/S0387-7604(87)80067-0. 3434722 10.1016/s0387-7604(87)80067-0

[19] Tariverdian G, Kantner G, Vogel F. A monozygotic twin pair with Rett syndrome. Hum Genet. 1987;75:88–90. 10.1007/BF00273849. 3804336 10.1007/BF00273849

[20] Partington MW. Rett syndrome in monozygotic twins. Am J Med Genet. 1988;29:633–7. 10.1002/ajmg.1320290322. 3377006 10.1002/ajmg.1320290322

[21] Kunio M, Yang C, Minakuchi Y, Ohori K, Soutome M, Hirasawa T, et al. Comparison of genomic and epigenomic expression in monozygotic twins discordant for Rett syndrome. PLoS ONE. 2013;8:e66729. 10.1371/journal.pone.0066729. 23805272 10.1371/journal.pone.0066729 PMC3689680

[22] Mittal K, Kabra M, Juyal R, BK T. De Novo deletion in MECP2 in a monozygotic twin pair: a case report. BMC Med Genet. 2011;12:113. 10.1186/1471-2350-12-113. 21871116 10.1186/1471-2350-12-113 PMC3176152

[23] Fehr S, Wilson M, Downs J, Williams S, Murgia A, Sartori S, et al. The CDKL5 disorder is an independent clinical entity associated with early-onset encephalopathy. Eur J Hum Genet. 2013;21:266–73. 10.1038/ejhg.2012.156. 22872100 10.1038/ejhg.2012.156 PMC3573195

[24] Mitter D, Pringsheim M, Kaulisch M, Plümacher KS, Schröder S, Warthemann R, et al. FOXG1 syndrome: genotype-phenotype association in 83 patients with FOXG1 variants. Genet Med. 2018;20:98–108. 10.1038/gim.2017.75. 28661489 10.1038/gim.2017.75

[25] Allen RC, Zoghbi HY, Moseley AB, Rosenblatt HM, Belmont JW. Methylation of HpaII and HhaI sites near the polymorphic CAG repeat in the human androgen-receptor gene correlates with X chromosome inactivation. Am J Hum Genet. 1992;51:1229–39. 1281384 PMC1682906

[26] Lau AW, Brown CJ, Peñaherrera M, Langlois S, Kalousek DK, Robinson WP. Skewed X-Chromosome inactivation is common in fetuses or newborns associated with confined placental mosaicism. Am J Hum Genet. 1997;61:1353–61. 10.1086/301651. 9399909 10.1086/301651 PMC1716095

[27] Perego S, Alari V, Pietra G, Lamperti A, Vimercati A, Camporeale N, et al. Modeling RTT syndrome by iPSC-Derived neurons from male and female patients with heterogeneously severe Hot-Spot MECP2 variants. Int J Mol Sci. 2022;23:14491. 10.3390/ijms232214491. 36430969 10.3390/ijms232214491 PMC9697612

[28] Cuddapah VA, Pillai RB, Shekar KV, Lane JB, Motil KJ, Skinner SA, et al. Methyl-CpG-binding protein 2 (MECP2) mutation type is associated with disease severity in Rett syndrome. J Med Genet. 2014;51:152–8. 10.1136/jmedgenet-2013-102113. 24399845 10.1136/jmedgenet-2013-102113 PMC4403764

[29] Vignoli A, La Briola F, Peron A, Turner K, Savini M, Cogliati F, et al. Medical care of adolescents and women with Rett syndrome: an Italian study. Am J Med Genet A. 2012;158A:13–8. 10.1002/ajmg.a.34367. 22139899 10.1002/ajmg.a.34367

[30] Hagberg B, Witt-Engerström I. Rett syndrome: epidemiology and nosology — progress in knowledge 1986 — A conference communication. Brain Dev. 1987;9:451–7. 10.1016/S0387-7604(87)80062-1. 3324795 10.1016/s0387-7604(87)80062-1

[31] Coleman M, Brubaker J, Hunter K. Rett syndrome: a survey of North American patients. J Intellect Disabil Res. 1988;32:117–24. 10.1111/j.1365-2788.1988.tb01397.x. 10.1111/j.1365-2788.1988.tb01397.x 3398038

[32] Tariverdian G. Follow-up of monozygotic twins concordant for the Rett syndrome. Brain Dev. 1990;12:125–7. 10.1016/s0387-7604(12)80192-6. 2344007 10.1016/s0387-7604(12)80192-6

[33] Migeon BR, Dunn MA, Thomas G, Schmeckpeper BJ, Naidu S. Studies of X inactivation and isodisomy in twins provide further evidence that the X chromosome is not involved in Rett syndrome. Am J Hum Genet. 1995;56:647–53. 7887418 PMC1801188

[34] Bruck I, Philippart M, Giraldi D, Antoniuk S. Difference in early development of presumed monozygotic twins with Rett syndrome. Am J Med Genet. 1991;39:415–7. 10.1002/ajmg.1320390411. 1715129 10.1002/ajmg.1320390411

[35] Subramaniam B, Naidu S, Reiss AL. Neuroanatomy in Rett syndrome. Neurology. 1997;48:399–407. 10.1212/WNL.48.2.399. 9040729 10.1212/wnl.48.2.399

[36] Ogawa A, Mitsudome A, Yasumoto S, Matsumoto T. Japanese monozygotic twins with Rett syndrome. Brain Dev. 1997;19:568–70. 10.1016/s0387-7604(97)00084-3. 9440804 10.1016/s0387-7604(97)00084-3

[37] Zhang X, Bao X, Zhang J, Zhao Y, Cao G, Pan H, et al. Molecular characteristics of Chinese patients with Rett syndrome. Eur J Med Genet. 2012;55:677–81. 10.1016/j.ejmg.2012.08.009. 22982301 10.1016/j.ejmg.2012.08.009

[38] Einspieler C, Marschik PB, Domingues W, Talisa VB, Bartl-Pokorny KD, Wolin T, et al. Monozygotic twins with Rett syndrome: phenotyping the first two years of life. J Dev Phys Disabil. 2014;26:171–82. 10.1007/s10882-013-9351-3. 29769795 10.1007/s10882-013-9351-3 PMC5951272

[39] Titlestad KB, Eldevik S. Brief report: modest but clinically meaningful effects of early behavioral intervention in twins with Rett Syndrome—A case study. J Autism Dev Disord. 2019;49:5063–72. 10.1007/s10803-019-04185-9. 31432309 10.1007/s10803-019-04185-9

[40] Carter JC, Lanham DC, Pham D, Bibat G, Naidu S, Kaufmann WE. Selective cerebral volume reduction in Rett syndrome: a multiple-approach MR imaging study. AJNR Am J Neuroradiol. 2008;29:436–41. 10.3174/ajnr.A0857. 18065507 10.3174/ajnr.A0857 PMC4403765

[41] Ishii T, Makita Y, Ogawa A, Amamiya S, Yamamoto M, Miyamoto A, et al. The role of different X-inactivation pattern on the variable clinical phenotype with Rett syndrome. Brain Dev. 2001;23:S161–4. 10.1016/S0387-7604(01)00344-8. 11738865 10.1016/s0387-7604(01)00344-8

[42] Cheadle JP. Long-read sequence analysis of the MECP2 gene in Rett syndrome patients: correlation of disease severity with mutation type and location. Hum Mol Genet. 2000;9:1119–29. 10.1093/hmg/9.7.1119. 10767337 10.1093/hmg/9.7.1119

[43] Girard M, Couvert P, Carrié A, Tardieu M, Chelly J, Beldjord C, et al. Parental origin of de Novo MECP2 mutations in Rett syndrome. Eur J Hum Genet. 2001;9:231–6. 10.1038/sj.ejhg.5200618. 11313764 10.1038/sj.ejhg.5200618

[44] Trappe R, Laccone F, Cobilanschi J, Meins M, Huppke P, Hanefeld F, et al. MECP2 mutations in sporadic cases of Rett syndrome are almost exclusively of paternal origin. Am J Hum Genet. 2001;68:1093–101. 10.1086/320109. 11309679 10.1086/320109 PMC1226090

[45] Xiol C, Vidal S, Pascual-Alonso A, Blasco L, Brandi N, Pacheco P, et al. X chromosome inactivation does not necessarily determine the severity of the phenotype in Rett syndrome patients. Sci Rep. 2019;9:11983. 10.1038/s41598-019-48385-w. 31427717 10.1038/s41598-019-48385-w PMC6700087

